# Environmental and Health Risks of Pesticide Use in Ethiopia

**DOI:** 10.5696/2156-9614-11.30.210601

**Published:** 2021-05-28

**Authors:** Beyene Negatu, Sisay Dugassa, Yalemtshay Mekonnen

**Affiliations:** 1 Pesticide Registration and Control, Ministry of Agriculture, Addis Ababa, Ethiopia; 2 Aklilu Lemma Institute of Pathobiology, Addis Ababa University, Addis Ababa, Ethiopia; 3 College of Natural and Computational Sciences, Addis Ababa University, Ethiopia

**Keywords:** acute poisoning, occupational exposure, fish, residue, pollution, DDT, soil, surface water, pesticides, breast milk

## Abstract

**Background.:**

There are frequent reports of unsafe pesticide use in many parts of Africa. Ethiopia is the second most populous nation in Africa with around 80% of the population still depending on agriculture which intensively uses pesticides. A number of studies have examined pesticide-related health and environmental risks in Ethiopia. However, most of these studies have been small in scale and it is therefore challenging to get a general overview of the extent of health risks and level of environmental contamination in the country.

**Objectives.:**

The aim of the present study was to synthesize and summarize contemporary knowledge on pesticide-related risks and relevant gaps in Ethiopia.

**Methods.:**

An electronic database search and gathering of grey literature were done to collect information on the risks of pesticide use in Ethiopia. The electronic search was conducted using MEDLINE (via PubMed) without any publication date or language specifications. The Preferred Reporting Items for Systematic Reviews and Meta Analyses (PRISMA) checklist was used as guide in the creation of this review.

**Discussion.:**

A synthesis of the reviewed studies showed evidence of health risks due to occupational pesticide exposure, surface water pollution with pesticides that could cause chronic health risks to the public, evidence of pesticide contamination of the environment (e.g., soil organisms, fish, bee colonies and wildlife) and local as well as international consumer risks due to pesticide residues in food items. In addition, there have been frequent reports of health and environmental hazards in association with cut-flower farms. There is also evidence of direct use of DDT (dichlorodiphenyltrichloroethane) on food crops and detection of DDT residues in surface water, soil and human breast milk. Those reported risks might be due to lack of knowledge among farm workers, negligence of farm owners, absence of post-registration monitoring systems and poor implementation of both national and international regulations in Ethiopia due to poor institutional capacity.

**Conclusions.:**

The health and environmental risks of inappropriate use of pesticides requires action by all concerned bodies. Improved institutional arrangements for enforcement of regulations, awareness and further intervention studies could lessen the high risks of pesticide misuse.

**Competing Interests.:**

The authors declare no competing financial interests.

## Introduction

Pesticides are crucial in contemporary agriculture, as without their use roughly up to 50% of crops could be lost in tropical warm climate countries.^1^ Nevertheless, pesticides have an intrinsic toxicity to non-target organisms in the environment, including human beings.^2^–^3^ The health effects from pesticide exposure are dependent upon the nature of the pesticide as well as the frequency, duration and intensity of exposure and individual susceptibility.^4^–^5^ In comparison to open farms, closed farms (greenhouses) have an extremely high risk of occupational exposure to pesticides that might be due to an enclosed area with relatively higher immediate humidity and temperature.^6^ In general, higher amounts of pesticide exposure within a short time (occupational exposure) may be followed by acute pesticide poisoning while recurrent exposures over a long period of time (exposure via pesticide residues in food items) may lead to chronic effects such as malignancy. Different studies have indicated various chronic health effects of pesticide exposure including effects on the nervous system (increased neurological symptoms and Parkinson's disease), respiratory system (respiratory symptoms and reduction in lung capacity), reproductive and endocrine disrupting effects (birth defects, menstrual cycle disruption and miscarriage) and are also linked to different types of malignancy (brain, breast, lymphoma and lung).^7^–^12^

Another health effect of unsafe pesticide use is acute pesticide poisoning, which is a significant problem in low- and middle-income countries (LMIC). The World Health Organization (WHO) has estimated there are between one to five million cases of pesticide poisoning per year, resulting in 20 000 fatalities among agricultural workers around the world,^13^ of which over 95% of the cases occur in LMIC even though fewer than 40% of the pesticides produced globally are used in LMIC.^14^ In addition to direct health risks, inappropriate use and management of pesticides could affect other non-target organisms and the natural environment (e.g., water, soil). Water, soil and air serve as important media for transportation of pesticides from one site to another. Pesticides can enter water bodies surrounding fields via spray drift, evaporation and deposition, and after rain events as runoff and erosion or drainage.^15^ Contamination of water bodies is a major concern for fish and other aquatic organisms such as mussels, oysters, prawns and lobsters.^16^–^17^

Pesticide contamination is a serious problem for ecosystems and harmful for all associated organisms, bees and wildlife, the indigenous microorganisms of soil, and the soil ecosystem.^18^ Pesticides can have teratogenic effects on vertebrates, including mammals, birds, reptiles, amphibians, and fish.^19^

The intensifying use of pesticides in modern agriculture has considerably improved food production. However, inappropriate pesticide use has increased residues of pesticides in food (plant and animal origins), which could be a risk to consumer health.^20^–^21^ Among different classes of pesticides, organochlorine pesticides pose the greatest concern. These chemicals are referred to as persistent organic pollutants (POPs) because of their long-term stability, lipophilicity, and their tendency to accumulate in the environment and in living organisms. The presence of a diverse range of persistent pesticide residues in the natural environment is of concern due to their ability to bio-concentrate and bio-accumulate in the food chain, and their resulting long-term impact on ecosystem integrity.^22^ This environmental contamination can lead to human exposure through consumption of residues of pesticides in food and drinking water.^23^

**Abbreviations**
*API*: Acute pesticide intoxication
*MRL*: Maximum residue limit

In sub-Saharan Africa countries, there have been many reports of unsafe use, handling, management, and disposal of pesticides due to lack of pesticide hazard related knowledge and training,^24^–^27^ increasing environmental and health risks. Organophosphate pesticides have been detected in food stuffs at levels dangerous to human health in Kenya.^28^ A study in Nigeria found that pesticide concentrations in bean samples exceeded their maximum residue limits.^29^ Respiratory, skin, joints and bones, and nervous system symptoms have been reported in individuals residing near horticultural farms due to pesticide exposure in Kenya.^30^ Spontaneous miscarriage and infant death have been associated with pesticide exposure among female South African small-scale farmers.^31^ Organochlorine pesticide residues have been found in water and sediment from Lake Victoria, Kenya.^32^ High levels of organochlorines have been reported in human blood sampled from residents in traditional rain-fed areas in Sudan.^33^ Strong associations have been found between neurological symptoms and past organophosphate poisoning in South African farmers.^34^ In addition, a large-scale panel survey in four African countries concluded that pesticide use is strongly correlated with higher costs associated with human illness, including increased health expenditures and time lost from work due to sickness in the recent past and suggested further targeted studies.^35^

In light of these reported pesticide-related risks in sub-Saharan Africa, there are institutional and legal arrangements for pesticide registration and post-registration monitoring activities. For example, the Kenyan Pest Control Products Board (PCPB) is an independent institute that regulates the registration, importation, exportation, manufacturing, distribution, and post-registration use of pesticides. Pesticide-related activities of the PCPB are supported by laboratories and at least six pieces of legislation related to pesticide usage (e.g., regulations on licensing of premises, registration, disposal, import and export).^36^ Similarly, the Tropical Pesticides Research Institute (TPRI) is an independent institute that registers and follows the post-registration of pesticides in Tanzania. The institute has facilities to undertake post-registration monitoring of pesticides including pesticide toxicology and environmental pollution in addition to training and services on management of pesticides. In addition, there is legislation supporting pesticide registration and post-registration activities in Tanzania (e.g., Plant Protection Act 1997 and Plant Protection Regulations 1998).^37^

Ethiopia is the second most populous nation in Africa with a population currently estimated at 115 million. In Ethiopia agriculture constitutes around 85% of the work force, comprised of mainly small-scale farmers and large-scale commercial farms that frequently use pesticides to increase agricultural productivity.^38^ However, similar to other sub-Saharan Africa countries, there are reports of unsafe pesticide use that have been associated with health and environmental risks in Ethiopia.^39^–^40^ To the best of our knowledge, no previous study has systematically summarized the overall extent of pesticide-related risks in Ethiopia. Therefore, the main objective of this review was to synthesize and summarize the current knowledge on contemporary pesticide-related risks and relevant gaps for future studies and the creation of appropriate education and mitigation measures by policy makers.

## Methods

In order to assess the risks of pesticides in Ethiopia, an electronic database search of published manuscripts and review of Gray literature i.e., unpublished official records and documents was performed. The Preferred Reporting Items for Systematic Reviews and Meta Analyses (PRISMA) checklist was used as a guide for the present review.^41^ In order to improve the quality of studies included in the present study, a standardized searching strategy, inclusion and exclusion criteria were used.

### Search strategy

The electronic literature search was conducted without any publication date or language specifications. For the literature research, MEDLINE (via Pub Med) was used with the following search strategy: (Pesticides) AND (exposure OR risk OR use) AND (health or environment) AND (Ethiopia). Moreover, the reference lists of eligible studies were checked for additional articles. The electronic databases search was performed in July 2020.

### Inclusion and exclusion criteria

Following the electronic database search and identification of applicable gray literature, documents were included in the review if they satisfied the following criteria: (1) categorized as original articles, (2) published in a peer-reviewed journal for published articles, or (3) if the exposure of interest (i.e., pesticides) and the outcomes of interest (pesticide-related risks) were included (clearly recorded) in the gray literature documents.

The exclusion criteria were documents (1) categorized as reviews or editorials for published articles, (2) in which the exposure of interest i.e., pesticide was not included or clearly recorded (3), or in which the outcomes of interest (pesticide-related risks) were not included or clearly recorded in the documents.

## Results

**Figure 1:**
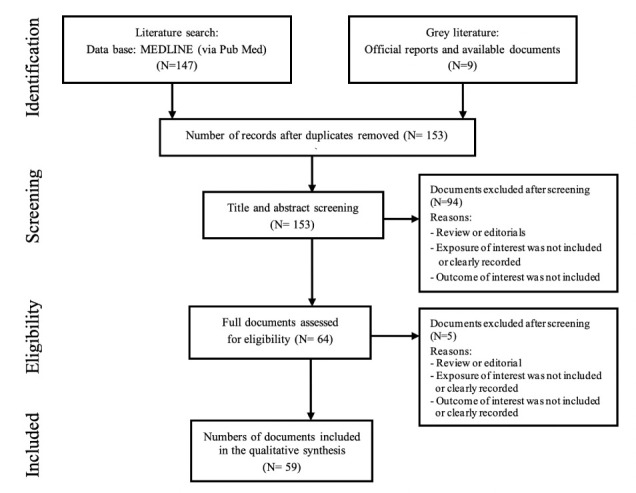
PRISMA flow diagram showing the study selection

A total of 147 relevant documents were identified via the electronic search and in addition nine documents were accessed from different governmental institutions. After clarification of duplication, 153 documents remained for screening based on document title and abstract. Sixty-four (64) potential documents for inclusion were identified after screening titles and abstracts. Finally, 59 documents were included in the review after the full texts of the documents were read *(Figure 1).*

Table 1 summarizes the results of the review by main categories of pesticide use, handling and management, health related risks, surface water related risks, fish-related risks, risks to honeybee colonies, risks to soil and wild animals, and pesticide residues in food.

**Table 1:** Included Studies by Review Category

**Review category**	**References**
Pesticide use and management	Negatu *et al.,* 2016,^42^ Mekonnen and Agonafir, 2002,^43^ Mekonnen and Agonafir, 2004,^44^ Karunamoorthi *et al.,* 2011,^45^ Mequanint *et al.,* 2019,^46^ Mengistie *et al.,* 2015,^47^ Gesesew *et al.,* 2016,^48^ Sahilu, 2016,^49^ Mengistie *et al.,* 2016,^50^ Mormeta, 2019.^51^
Health risks	Negatu *et al.,* 2018,^52^ Ejigu and Mokonnen, 2005,^53^ Hanssen *et al.,* 2015,^54^ Mekonnen and Agonafir, 2002,^55^ Mekonnen and Agonafir, 2004,^56^ Negatu *et al.,* 2016,^57^ Fix *et al.,* 2020,^58^ Getu, 2009,^59^ Shentema *et al.,* 2020,^60^ Gezmu, 2013,^61^ Atkure and Ahmed, 2013,^62^ Sahilu, 2016,^49^ Mormeta, 2019,^51^ Nigatu, 2016,^63^ Tamirat, 2007,^64^ Tefera *et al.,* 2019,^65^ Abula and Wondmikun, 2006,^66^ Abebe, 1991,^67^ Desalew 2011,^68^ Azazh, 2011,^69^ Nigatu *et al.,* 2016.^70^
Surface water risks	Shegen *et al.,* 2016,^71^ Teklu *et al.,* 2016,^72^ Jansen and Harmsen *et al.,* 2011,^73^ Tamirat, 2007,^64^ Mellese, 2016,^40^Teklu *et al.,* 2016.^74^
Risks to fish	Deribe *et al.,* 2014,^75^ Yohannes *et al.,* 2013,^76^ Yohannes *et al.,* 2014,^77^ Deribe *et al.,* 2011.^78^
Risk to bee colonies	Melisie *et al.,* 2016,^79^ Belie *et al.,* 2009,^80^ Desalgn, 2014,^81^ Mengistu and Beyene, 2014,^82^ Mekonnen *et al.*, 2018 83 Werkneh, 2011,^84^
Risks to soil and wildlife	Hussen *et al.,* 2007,^85^ Yohannes *et al.,* 2014.^77^
Pesticide residues in food	Daba *et al.,* 2011,^86^ Mekonen *et al.,* 2014,^87^ Zelelew *et al.,* 2018,^88^ Mekonen *et al.,* 2015,^89^ Mekonen *et al.,* 2017,^90^ Fesseha *et al.,* 2020,^91^ Deti *et al.,* 2014,^92^ Gebremichael *et al.,* 2016,^93^ Letta and Attah, 2013,^94^ Mulugeta *et al.,* 2017.^95^

### Pesticides use, handling, and management

In Ethiopia, pesticides are mainly imported for agricultural purposes, and lesser amounts of pesticides are imported for health care (vector control) and industrial purposes. Chemical pesticide use in Ethiopia has been historically low, but due to recent developments in intensification and expansion of modern agricultural activities including commercial horticultural farms such as small-scale irrigated farms, large-scale open farms and cut-flower greenhouses, there has been almost a threefold increase within a decade (1440 to 4586 tons from 2001 to 2013).^42^

Surveys conducted before 2011 on pesticide-related knowledge and practices in Ethiopia have indicated that farm workers had limited knowledge on proper pesticide use and handling, have inadequate awareness of safe pesticide management, and exercise poor hygienic and sanitation practices.^43^–^45^ Furthermore, other relatively recent studies have indicated similar results to previously conducted surveys which indicate ongoing improper pesticide use (misuse, incompatible pesticide mixing, over spraying of crops), pesticide handling (inadequate use of personal protective equipment (PPE)) and pesticide management such as empty pesticide burial/burning and disposing in nearby farm fields.^46^–^48^ In addition, studies in Ethiopia have indicated the continued use of a POPs such as dichlorodiphenyltrichloroethane (DDT) directly on food crops.^46^, ^48^–^49^

In addition to absence of knowledge in pesticide users (i.e. farmers and farm workers) leading to improper pesticide use and management, there have been reports of private actors such as retailers and state actors recommending inappropriate management of pesticides, including burning or burying of empty packages.^50^ Similarly, a study on governmental agricultural extension workers in Ethiopia indicated the surveyed workers have inadequate knowledge of pesticide-related hazards and may recommend improper handling of pesticides to farmers.^51^

### Health risks

As previously explained, there has been intensification in pesticide use accompanied by unsafe handling and management of pesticides in Ethiopia. This situation has resulted in pesticide exposure episodes leading both to acute and chronic health effects in Ethiopian farmers and farm workers. Regarding acute pesticide poisoning in Ethiopia, a standardized survey using a WHO case definition of severe forms of acute pesticide poisoning indicated a 16% overall prevalence of severe acute pesticide poisoning (including discontinuing work and/or fainting while applying pesticides).^52^ In addition, the highest prevalence (32%) was recorded among applicators in cut-flower greenhouses, and the same study also showed a “healthy worker selection effect”, i.e. a decrease of acute pesticide poisoning risk with years of service that might indicate that poisoned farm workers leave their employment after severe acute poisoning incidents.^52^

Respiratory health is the most frequently studied occupational health effect of pesticide exposure in Ethiopia; different studies have indicated higher prevalence of respiratory symptoms^53^–^54^ and reductions in respiratory function in individuals occupationally exposed to pesticides.^44^,^55^ With the exception of Hanssen *et al.*, all the other studies were done primarily among male applicators and in former state farms (i.e., large-scale open farms).^54^ However, another relatively larger study that focused both on male pesticide applicators as well as female re-entry workers in commercial farming systems in Ethiopia (i.e., small-scale irrigated farms, large-scale open farms and cut-flower greenhouses) indicated significant exposure-response associations of occupational pesticide exposure with respiratory symptoms and reductions in lung function.^56^–^57^ Negatu *et al.* compared the magnitude of the reduction in lung function due to pesticide exposure with a standardized estimate of lung function loss due to cigarette smoking per year and the observed effect due to pesticide exposure was 3- and 5-fold greater per year than cigarette smoking in males and females, respectively.^56^

Other than some of the aforementioned studies on the health effects of pesticides that include cut-flower greenhouses farms^52^,^54^,^56^ some studies specifically on workers from cut-flower greenhouses farms in Ethiopia have indicated diverse health problems including swelling of the feet and kidney problems.^58^ A high prevalence of abnormal serum cholinesterase levels, respiratory and dermal symptoms have been reported.^54^,^59^ In addition, there have been many reports^60^–^63^ of the negative health and environmental impacts of cut-flower greenhouses farms such as floriculture farms in Ethiopia. In cut-flower greenhouses farms, in comparison to large-scale open farms or small-scale irrigated farms, there are reports of relatively higher (8–13-fold) intensity of pesticide use, use of unregistered pesticides, use of WHO highly hazardous pesticide list pesticides, and higher occupational pesticide exposures compared with open field farming that might lead to higher frequency of health symptoms.^42^,^45^,^63^–^64^

The other health-related pesticide risks in Ethiopia include self-poisoning and residential pesticides exposure risks. There are many hospital-based studies that indicate pesticides as the main means of intentional self-poisoning.^65^–^68^ The high frequency of suicide using pesticides may be due to easy availability of highly toxic but cheap pesticides such as organophosphates in illegal (open) markets. To our knowledge there is only one study in Ethiopia that has investigated the association of acute pesticide intoxication (API) with residential proximity to green houses. It indicated that 42% of those residing close to flower farms (<5 km) are reported to have experienced API, compared to 11% of those living farther away (5–12 km) with a significant prevalence ratio (PR) of (PR=3.7, 95% CI: 2.6–5.4).^69^

### Surface water risks

Pesticide contamination of water bodies can be hazardous both directly and indirectly to humans and other organisms that live near water. A study conducted in surface water samples around western Ethiopia and Addis Ababa (the capitol) showed mean concentrations of 2, 4-D, malathion, diazinon and fenpropimorph ranging from 1.59–13.90 μg/l and 0.11–138 μg/l for Jimma and Addis Ababa water samples, respectively.^70^ The same study indicated the residue levels of some of the pesticides were above the European drinking water guideline values. The study also indicated a clear chronic risk to public health, particularly from exposure of diazinon and fenpropimorph due to higher estimated daily intake (EDI) than the acceptable daily intake (ADI) of these pesticides.^70^

Environmental monitoring studies in the Ethiopian Lake Zeway area showed a higher chronic risk posed by the insecticide spiroxamine (using the European standardized cut-off value of pesticide residues of 0.1 μg/L) if surface water is used for drinking purposes^71^ and higher acute exposure toxicity ratio values for pesticides clofentezine, sulfur, spiroxamine and methomyl that can pose an acute toxic risk to aquatic organisms.^72^ Additional surface water pesticide risk assessment studies in the Debre Zeit area of central Ethiopia indicated that lambda-cyhalothrin, endosulfan, profenofos, and diazinon pesticides may pose high risks to the aquatic ecosystem^73^ and a decrease of macro invertebrate biodiversity and disappearance of sensitive taxa may be due to chemical pesticide loads.^63^ Similar to the negative health risk posed by cut-flower greenhouses farms as discussed in the health risk section above, there are many studies on the negative impacts of cut-flower greenhouses farms on surface water, as risk assessment studies^63^,^72^–^73^ include surface water samples of nearby effluents of floriculture farms, indicating a risk to public health and aquatic systems.

### Risks to fish

There are few pesticide-related risk assessment studies on fish in Ethiopia. A study on tissue samples of fish collected from Lake Hawassa in southern Ethiopia showed contamination with organochloride pesticides (OCPs) including DDT and endosulfan. In addition to the risk posed by OCPs to fish, the study also indicated a risk to consumers' health, in particular for children between the ages of 0–1 year.^74^ Another study on samples of muscle and liver of three fish species from Lake Hawassa indicated residues of DDTs that could have biomagnified in the lake's food web.^75^ Similar studies in lakes of the Central Rift Valley, Ethiopia indicated OCP contamination in muscle samples of five fish species from Lake Zeway^76^ and residues of DDT, endosulfans and chlorpyrifos from Lake Koka.^77^ In addition, the study on Lake Koka indicated bio magnification of DDTs in the food web, similar to the results of the Lake Hawassa study.^75^

### Risk to bee colonies

Many studies have been conducted on the effects of pesticide use in bee colonies across Ethiopia, for example a study in the Ethiopian Central Rift Valley where pesticides are used intensively for small-scale horticultural production indicated that 48.3% of beekeepers abandoned beekeeping as a result of colony losses due to pesticide applications.^78^ Similarly, studies in other parts of Ethiopia, including the Enebse and Bure districts,^79^ the Dangila, Guangua and Mecha districts,^80^ the Gojjam zone of northwest Ethiopia,^81^ the Ejere District of western Ethiopia,^82^ and others^83^ reported a decreasing trend of honeybee populations and their products due indiscriminate pesticide application.

### Risks to soil and wildlife

A study in Ethiopia^84^ around Upper Awash agriculture industry enterprises detected substantial amount of OCPs (i.e., sigma endosulfans up to 56000 and sigma DDTs up to 230 ng g (-1) dry weight) which could be a threat to the surrounding and downstream ecosystems. In addition, the only study on wild birds in the Ethiopian Rift Valley region indicated the main DDT metabolite, p,p'-DDE, was most abundant and significantly greater concentrations in the investigated bird species (up to 138.5 μg/g lipid), that could have deleterious effects on survival and/or reproduction of birds.^76^

### Pesticide residues in food

There have been many risk assessment studies of pesticide residues in plant products in Ethiopia. All of the assessments in Ethiopia detected pesticide residues in samples and some were above the allowed maximum residue limits (MRLs). For example, diazinon residues were detected in wheat samples.^85^ All food items scrutinized for residues contained one or more pesticide residues.^86^ Analyses for 2, 4-D, aldrin, endosulfan and DDT pesticides in commercially available wheat samples showed detectable residues^89^ and all maize samples showed contamination by DDT.^88^ However, all of the above studies indicated a detectable residue lower than MRL except Mekonen *et al.* in which more than 33% of the food samples were above MRLs and Mekonen *et al.* where mean concentrations of DDT in maize samples were far above the MRLs.^86^,^88^ Additionally, an investigation of pesticide residues in khat, a common stimulant used in Ethiopia, indicated that 80% of the khat samples contained DDT and some of the residues were above MRLs.^89^

In addition to the risk of pesticide residues in plant products, other studies have shown contamination and risk of pesticide residues in animal food products. A study in southern Ethiopia indicated 60% of the dairy farm owners offer or sell products to the public from animals treated with a variety of drugs, including pesticides, without a withdrawal period for the drug, which poses a risk to consumers' health.^90^ Similar studies detected varying levels of persistent organochlorine pesticides residues from cow and goat milk.^91^ In addition, a 3-fold higher DDT residue concentration above the acceptable daily intake set by the WHO was detected in human mothers' and cows' milk samples.^92^ On top of the risk of pesticide residues in dairy products, other studies in Ethiopia also showed residues of organochloride pesticides in cattle carcasses^93^ and honey samples.^94^

## Discussion

Pesticides have the benefit of improving agricultural yields, however, they also pose environmental as well as short- and long-term health concerns globally. This review presents a summary of the overall results of published studies on the environmental and health risks of pesticides in Ethiopia. Higher risk of health effects due to occupational pesticide exposure has been reported in farm workers, and this may lead to the early retirement of young (productive) farm workers. Surface water pollution from pesticides presents chronic health risks to the public and endangers aquatic ecosystems. Other studies point to the risks of pesticides to non-target organisms such as soil organisms, bee colonies and terrestrial birds. Pesticide residues in both plant and animal origin foods are another risk posed to public health in Ethiopia and countries which import these agricultural products. Some pesticides are characterized by higher residues. For example, DDT (a persistent bio-accumulative pesticide) is a frequently reported pesticide with higher residues in food items in Ethiopia. This indicates even higher risks to infants and children who are highly susceptible to pesticide toxicity due to their stage of development as well as lower body weight. In addition to local consumer risks from pesticide residues, official reports and documents from the Ethiopian Ministry of Agriculture (MOA) (unpublished, Export rejection notification summary. MOA. Addis Ababa, Ethiopia. 2013; Export rejection notification summary. MOA. Addis Ababa, Ethiopia. 2015) have indicated incidents of the border rejection of exported crop products at international markets, mainly at European borders *(Table 2).*

**Table 2:** Rejection of Crop Exports from Ethiopia Due to Unacceptable Pesticide Residues

**Crop**	**Year**	**Country**	**Pesticide**
Coffee	2008	Japan^97^	Organochlorides
Beans	2013	Spain^97^	Malathion and diazinon
Sesame	2014	Japan^98^	2-4-D
Mug beans	2014	Italy^98^	Malathion
White pea beans	2014	Italy^98^	Malathion
Dried white beans	2014	Italy^98^	Fenthion and malathion
Kidney beans	2014	Italy^98^	Diazinon
Mung beans	2015	Italy^98^	Malathion
Beans	2015	Italy^98^	Propoxur

The first official export rejection due to high pesticide residues occurred in Japan for Ethiopian coffee beans in 2008 (*Table 2*). Subsequently, Ethiopian coffee bean imports were banned for two years, creating panic among local coffee producers as well as other importing countries. After this incident, Ethiopia established its first pesticide residue laboratory for coffee. However, as the official report from the MOA indicated, the border rejection of Ethiopian export crops continued and increased in frequency and range of affected crop (*Table 2*). None of the pesticides listed in Table 2 are registered to be used for their corresponding registration purpose (i.e., a pesticide is usually registered to be used for a specific target pest and crop) under the Ethiopian pesticide registration system. For example, malathion is not registered to be used on pulses (mung beans, white pea beans, dried white beans, or dried mung beans). Diazinon, propoxur and fenthion are also not registered to be used on pulses. Propoxur is not registered to be used in crop protection, rather it is a public health pesticide used for indoor residual spraying to control malaria (Unpublished list of pesticides registered in Ethiopia. MOA. Addis Ababa, Ethiopia. 2016). Similarly, diazinon is also commonly used for the control of external parasites in animals (Unpublished list of pesticides registered in Ethiopia. MOA. Addis Ababa, Ethiopia. 2016).

There are other additional relevant issues in association with pesticide risks in Ethiopia. There is evidence of direct use of DDT on food crops as well as detection of DDT in surface water, soil, food items and the human body (breast milk). As DDT is a persistent pesticide that could bioaccumulate in the food chain, there might be a risk of long-term impact on human health as well as the ecosystem. The other important issue involves the relatively higher health problems and environmental risks reported in association with Ethiopian greenhouse flower farms. This suggests the need for intervention studies on environmental and health risks in these farms along with an economic assessment (cost-benefit analysis) to determine whether the national benefit of those farms outweighs their negative impacts.

In order to balance the profits of increased agricultural production and the risks of intensified pesticide use on the environment and health, there is a need for increased pesticide regulation. The first pesticide regulation in Ethiopia was authorized in 1990 through Special Decree No. 20/1990, which was followed by pesticide registration and control proclamation No. 674/2010, 95 which is the latest pesticide related regulation in Ethiopia. Additionally, Ethiopia is a signatory to many international conventions on the use of pesticides (e.g., the Stockholm Convention on Persistent Organic Pollutants).^96^ In Ethiopia, the responsibility for registration, control of import and distribution of pesticides is given to the MOA by Proclamation No. 674/2010.^95^

The proclamation also states that “any employer shall provide facilities and protective clothing for safe handling of pesticides, no person shall dispose of any pesticide in a manner that can harm human health and the environment and an inspector assigned by the ministry can carry out surveillance to insure conditions of registration are complied.”^95^ As pesticide registration and control are multispectral tasks, the proclamation indicated the need for a National Pesticide Advisory Board consisting of specialists from other relevant institutions, such as the Environmental Protection Authority (Ethiopia) and the MOH to advise the MOA on all relevant issues related to pesticides in Ethiopia. This board could advise registration and post-registration monitoring activities in Ethiopia, but the board is not currently operational.

The present review showed an increasing trend in pesticide use intensity and continued inappropriate use, handling and management of pesticides associated with environmental and health risks in Ethiopia. This might be due to insufficient knowledge of pesticide hazards among farmers on small-scale farms that might be attributable to lack of access to information and low literacy levels. Managers of large-scale and cut-flower greenhouses of pesticide hazard should provide appropriate pesticide risk reduction measures to their farm workers. Another issue highlighted by the studies in this review involves pesticide registration, as it was found that the controlling body for pesticide usage (the Ethiopian Ministry of Agriculture) usually does not coordinate with other concerned institutions such as the Ministry of Health and the Ethiopian environmental protection authority and do not perform post-registration follow-up of pesticide usage. This step is important to identifying unacceptable health and environmental risks of pesticides in actual field use. Therefore, there is no way of insuring that registered products are properly handled and managed in accordance with Ethiopian proclamation 765/2010.

In addition, coordinated work on the safe use of pesticides among all relevant governmental stakeholders (federal ministries, institutions, and regional states) as well as with non-governmental organizations working on similar activities is another gap highlighted in the studies in the present review. There is an absence of a pesticide monitoring system for both the environment and public health which would be an important to tool to detect early environmental and health risks due pesticide or usage so that appropriate regulatory measures could be taken (e.g., use restriction or banning of a hazardous pesticide). The last issue highlighted is the poor institutional capacity for implementation of both national and international regulations such as the Stockholm Convention on POPs in Ethiopia. Pesticide registration and control activities in Ethiopia are performed by a case team of around 18 workers and one laboratory (for detecting residues and pesticide formulation) which is not currently operational.

### Strengths and limitations

The strengths of the present review include a clearly stated objective, reproducible methodology with clear criteria of inclusion and exclusion, and additional searches for grey literature to minimize risk of publication bias. However, it did not use the full PRISMA statement checklist (e.g. we did not systematically assess the risk of bias in each of the identified studies) as most of the checklist items are specifically relevant for systematic reviews of non-randomized studies assessing the benefits and harms of interventions.^40^ In addition, due to limited data on the border rejection of crop exports it was not possible to determine the exact level of contamination of export crops by pesticides or risk to international consumers.

## Conclusions

Studies in the present review indicate that injudicious use of pesticides has resulted in serious environmental and public health risks. Health risks arise due to occupational exposures, pesticide residues in food items, and contact with sprayed surfaces, while environmental risks occur due to surface water pollution, pesticide drift in the environment, and excessive pesticides applied on treatment areas. Those risks are mainly attributable to poor institutional capacity to undertake post-registration monitoring of pesticides and lack of implementation of available pesticide legislation. There is also a great need to raise awareness of the public on issues of pesticide misuse.

In light of the reviewed studies and identified gaps there is a need of policy direction to establish an independent institution for pesticide registration and control activities in Ethiopia. This institution could coordinate pesticide registration, pesticide-related hazard extension information, post-registration environmental and health monitoring, and pesticide regulatory issues with appropriate trained staff and laboratory capacity, similar to other neighboring east African countries such as the Kenyan Pest Control Products Board and the Tropical Pesticides Research Institute of Tanzania. There is also a need for further intervention studies on the environmental and health risks of pesticide use in agricultural activities, such as cut-flower farms, before the occurrence of irreversible environmental and health impacts of pesticides.

## References

[1] Popp J, Peto K, Nagy J (2013). Pesticide productivity and food security. A review. Agron Sustain Dev.

[2] Grung M, Lin Y, Zhang H, Steen AO, Huang J, Zhang G (2015). Pesticide levels and environmental risk in aquatic environments in China--A review. Environ Int.

[3] Lerro CC, Koutros S, Andreotti G, Hines CJ, Blair A, Lubin J (2015). Use of acetochlor and cancer incidence in the Agricultural Health Study. Int J Cancer.

[4] Gomes J, Lloyd OL, Revitt DM (1999). The influence of personal protection, environmental hygiene and exposure to pesticides on the health of immigrant farm workers in a desert country. Int Arch Occup Environ Health.

[5] Sanborn M, Kerr KJ, Sanin LH, Cole DC, Bassil KL, Vakil C (2007). Non-cancer health effects of pesticides: systematic review and implications for family doctors. Can Fam Physician.

[6] Kundiev YuI, Krasnyuk EP, Viter VPh (1986). Specific features of the changes in the health status of female workers exposed to pesticides in greenhouses. Toxicol Lett.

[7] Alavanja MC, Bonner MR (2012). Occupational pesticide exposures and cancer risk: a review. J Toxicol Environ Health B Crit Rev.

[8] London L, Beseler C, Bouchard MF, Bellinger DC, Colosio C, Grandjean P (2012). Neurobehavioral and neurodevelopmental effects of pesticide exposures. Neurotoxicology.

[9] Murphy H The Long Term Effects of Pesticide Exposure on Human Health. https://www.Migrantclinician.org/files/ChronicHealthEffects_PesticidesPresentation_0.ppt.

[10] Hoppin JA, Umbach DM, London SJ, Lynch CF, Alavanja MC, Sandler DP (2006). Pesticides and adult respiratory outcomes in the agricultural health study. Ann N Y Acad Sci.

[11] Weselak M, Arbuckle TE, Wigle DT, Krewski D (2007). In utero pesticide exposure and childhood morbidity. Environ Res.

[12] Farr SL, Cooper GS, Cai J, Savitz DA, Sandler DP (2004). Pesticide use and menstrual cycle characteristics among premenopausal women in the Agricultural Health Study. Am J Epidemiol.

[13] Gunnell D, Eddleston M (2003). Suicide by intentional ingestion of pesticides: a continuing tragedy in developing countries. Int J Epidemiol.

[14] Jeyaratnam J (1990). Acute pesticide poisoning: a major global health problem. World Health Stat Q.

[15] Knauer K (2016). Pesticides in surface waters: a comparison with regulatory acceptable concentrations (RACs) determined in the authorization process and consideration for regulation. Environ Sci Eur.

[16] Essumang D, Chokky L (2009). Pesticide residues in the water and fish (Lagoon tilapia) samples from Lagoons in Ghana. Bull Chem Soc Ethiopia.

[17] Barizon RRM, Figueiredo RO, de Souza Dutra DRC, Regitano JB, Ferracini VL (2020). Pesticides in the surface waters of the Camanducaia River watershed, Brazil. J Environ Sci Health B.

[18] Sharma A, Kumar V, Shahzad B, Anveer M, Sidhu GPS, Handa N (2019). Worldwide pesticide usage and its impacts on ecosystem. SN Appl Sci.

[19] Garces A, Pires I, Rodrigues P (2020). Teratological effects of pesticides in vertebrates: a review. J Environ Sci Health B.

[20] Tago D, Andersson H, Treich N (2014). Pesticides and health: a review of evidence on health effects, valuation of risks, and benefit-cost analysis. Adv Health Econ Health Serv Res.

[21] Modi CM, Patel HB, Mody SK (2013). Animal husbandry practice to contaminants and residues of chemical in animal origin foods and health hazard. Int J Mol Vet Res.

[22] Madadi VO (2017). Occurrence and Distribution of Organochlorine Pesticide Residues in Water and Soil Samples from Kargi Area, Marsabit County, Kenya. Int J Sci Res Sci Eng Technol.

[23] Damalas CA, Eleftherohorinos IG (2011). Pesticide exposure, safety issues, and risk assessment indicators. Int J Environ Res Public Health.

[24] Ngowi AVF (2003). A study of farmers' knowledge, attitude and experience in the use of pesticides in coffee farming. The African Newsletter on Occupational Health and Safety.

[25] Ntow WJ, Gijzen HJ, Kelderman P, Drechsel P (2006). Farmer perceptions and pesticide use practices in vegetable production in Ghana. Pest Manag Sci.

[26] Williamson S, Ball A, Pretty J (2008). Trends in pesticide use and drivers for safer pest management in four African countries. Cro Prot.

[27] Naidoo S, London L, Rother HA, Burdorf A, Naidoo RN, Kromhout H (2010). Pesticide safety training and practices in women working in small-scale agriculture in South Africa. Occup Environ Med.

[28] Samuel N (2009). Evaluating farmers knowledge in pesticide handling and determining pesticide levels in maize after various processing methods and different storage conditions. https://ir-library.ku.ac.ke/bitstream/handle/123456789/646/Njoroge%20Samuel.pdf?sequence=3&isAllowed=y.

[29] Akinneye JO, Adedolapo AO, Adesina FP (2018). Quantification of Organophosphate and Carbamate residue on stored grains in Ondo State, Nigeria. J Biol Med.

[30] Tsimbiri PF, Moturi WN, Sawe J, Henley P, Bend J (2015). Health Impact of Pesticides on Residents and Horticultural Workers in the Lake Naivasha Region, Kenya. Occup Dis Environmen Med.

[31] Naidoo S, London L, Burdorf A, Naidoo R, Kromhout H (2011). Spontaneous miscarriages and infant deaths among female farmers in rural South Africa. Scand J Work Environ Health.

[32] Osoro EM, Wandiga SO, Abong'o DA, Madadi VO, Macharia JW (2016). Organochlorine Pesticides Residues in Water and Sediment from Rusinga Island, Lake Victoria, Kenya. IOSRJAC.

[33] Abdelbagi AO, Elbashir AB, Hammad AM, Elzorgani GA, Laing MD (2015). Organo chlorine levels in human blood from residents in areas of limited pesticide use in Sudan. Toxicol Environ Chem.

[34] London L, Nell V, Thompson M, Myers J (1998). Effects of long term organophosphate exposures on neurological symptoms, vibration sense and tremor among South African farm workers. Scand J Work Environ Health.

[35] Sheahan M, Barrett CB, Goldvale C (2017). Human health and pesticide use in Sub-Saharan Africa. Agricultural Economics.

[36] Pest Control Products Board (PCPB) https://www.pcpb.go.ke/.

[37] Lahr JR, Buij F. Katagira, van der Valk H Pesticides in the Southern Agricultural Growth Corridor of Tanzania (SAGCOT). A scoping study of current and future use, associated risks and identification of actions for risk mitigation.

[38] Ethiopia-Country Profile-2020 Accessed [2020 February 6]. https://www.indexmundi.com/ethiopia/.

[39] Negatu B (2017). Occupational risks and health effects of pesticides in three commercial farming systems in Ethiopia. https://dspace.library.uu.nl/bitstream/handle/1874/351047/Mormeta.pdf?sequence=1.

[40] Mellese BT (2016). Environmental risk assessment of pesticides in Ethiopia a case of surface water systems.

[41] Moher D, Liberati A, Tetzlaff J, Altman DG (2009). The PRISMA Group. Preferred Reporting Items for Systematic Reviews and Meta Analyses: The PRISMA Statement. PLoS Med.

[42] Negatu B, Kromhout H, Mekonnen Y, Vermeulen R (2016). Use of Chemical Pesticides in Ethiopia: a cross-sectional comparative study on Knowledge Attitude and Practice of farmers and farm workers in three farming system. Ann Occup Hyg.

[43] Mekonnen Y, Agonafir T (2002). Pesticide sprayers' knowledge, attitude and practice of pesticide use on agricultural farms of Ethiopia. Occup Med(Lond).

[44] Mekonnen Y, Agonafir T (2004). Lung function and respiratory symptoms of Pesticide sprayers in state farms of Ethiopia. Ethiop Med J.

[45] Karunamoorthi K, Mohammed A, Jemal Z (2011). Peasant Association Member's Knowledge, Attitudes, Practices towards Safe Use of Pesticide Management. Am J Ind Med.

[46] Mequanint C, Getachew B, Mindaye Y, Amare DE, Guadu T, Dagne H (2019). Practice towards pesticide handling, storage and its associated factors among farmers working in irrigations in Gondar town, Ethiopia. BMC Res Notes.

[47] Mengistie BT, Mol A, Oosterveer P (2017). Pesticide use practices among smallholder vegetable farmers in Ethiopian Central Rift Valley. Environ Dev Sustain.

[48] Gesesew HA, Woldemichael K, Massa D, Mwanri L (2016). Farmers Knowledge, Attitudes, Practices and Health Problems Associated with Pesticide Use in Rural Irrigation Villages, Southwest Ethiopia. (2016) PLoS One.

[49] Sahilu TA Stewardship towards Responsible Management of Pesticides The case of Ethiopian Agriculture. https://pub.epsilon.slu.se/13580/1/amera_t_160818.pdf.

[50] Mengistie BT, Mol AP, Oosterveer P (2016). Private environmental governance in the Ethiopian pesticide supply chain: importation, distribution and use. NJAS Wageningen J Life Sci.

[51] Mormeta BN (2019). Assessment of pesticide hazard related knowledge and practices of agricultural extension workers in selected small-scale horticulture production areas in Ethiopia. J Agri Environ Intern Develop.

[52] Negatu B, Vermeulen R, Mekonnen Y, Kromhout H (2018). Neurobehavioral symptoms and acute pesticide poisoning: a cross-sectional study among male pesticide applicators selected from three commercial farming systems in Ethiopia. Occup Environ Med.

[53] Ejigu D, Mekonnen Y (2005). Pesticide use on agricultural fields and health problems in various activities. East Afr Med J.

[54] Hanssen VM, Nigatu AW, Zeleke ZK, Moen BE, Bråtveit M (2015). High Prevalence of Respiratory and Dermal Symptoms among Ethiopian Flower Farm Workers. Arch Environ Occup Health.

[55] Mekonnen Y, Agonafir T (2002). Effects of pesticide application on respiratory health of Ethiopian farm workers. Int J Occup Environ Health.

[56] Negatu B, Kromhout H, Mekonnen Y, Vermeulen R (2017). Occupational pesticide exposure and respiratory health: a large-scale cross-sectional study in three commercial farming systems in Ethiopia. Thorax.

[57] Fix J, Annesi-Maesano I, Baldi I, Boulanger M, Cheng M, Cortes S (2020). Gender differences in respiratory health outcomes among farming cohorts around the globe: findings from the AGRICOH consortium. J Agromedicine.

[58] Getu M (2009). Ethiopian floriculture and its impact on the environment. Mizan Law Review.

[59] Shentema MG, Kumie A, Bråtveit M, Deressa W, Ngowi AV, Moen BE (2020). Pesticide Use and Serum Acetyl cholinesterase Levels among Flower Farm Workers in Ethiopia—A Cross-Sectional Study. Int J Environ Res Pub Heal.

[60] Gezmu AB (2013). The human impacts of flower farm development in the Ethiopian Rift Valley region.

[61] Atkure D, Ahemed A (2013). Occupational induced health problems in floriculture workers in Sebeta and surrounding areas, West Shewa, Oromia, Ethiopia. Ethiop J Heal Develop.

[62] Nigatu S (2016). On the social and environmental impacts of Tinaw floriculture industry in the surrounding community, Ezha Woreda, Guraghe zone southern nations nationalities and peoples region (SNNPR): Ethiopia. http://etd.aau.edu.et/handle/123456789/9855.

[63] Tamirat SM Assessment of the ecological impacts of floriculture industries using physico-chemical parameters and benthic macro invertebrates metric index along Wedecha River, Debrezeit, Ethiopia. http://etd.aau.edu.et/handle/123456789/7200?show=full.

[64] Tefera YM, Thredgold L, Pisaniello D, Gaskin S (2019). The greenhouse work environment: a modifier of occupational pesticide exposure?. J Environ Sci Heal B.

[65] Abula T, Wondmikun Y (2006). The pattern of acute poisoning in a teaching hospital, north-west Ethiopia. Ethiop Med J.

[66] Abebe M (1991). Organophosphate pesticide poisoning in 50 Ethiopian patients. Ethiop Med J.

[67] Desalew M, Aklilu A, Amanuel A, Addisu M (2011). Pattern of acute adult poisoning at Tikur Anbessa specialized teaching hospital, a retrospective study, Ethiopia. Hum Exp Toxicol.

[68] Azazh A (2011). Severe organophosphate poisoning with delayed cholinergic crisis, inter mediate syndrome and organophosphate induced delayed polyneuropathy on succession. Ethiop J Health Sci.

[69] Nigatu AW, Bråtveit M, Moen BE (2016). Self-reported acute pesticide intoxications in Ethiopia. BMC Public Health.

[70] Shegen SM, Argaw R, Simanesew A, Houbraken M, Senaeve D, Ambelu A, Spanoghe P (2016). Pesticide residues in drinking water and associated risk to consumers in Ethiopia. Chemosphere.

[71] Teklu BM, Hailu A, Wiegant DA, Scholten BS, Van den Brink PJ (2016). Impacts of nutrients and Pesticides from small and large scale agriculture on the water quality of Lake Ziway, Ethiopia. Environ Sci Pollut Res.

[72] Jansen HC, Harmsen J Pesticide monitoring in the Central Rift Valley 2009–2010. https://library.wur.nl/WebQuery/wurpubs/406938.

[73] Teklu BM, Adriaanse PI, Van den Brink PJ (2016). Monitoring and risk assessment of pesticides in irrigation systems in Debra Zeit, Ethiopia. Chemosphere.

[74] Deribe E, Rosseland BO, Borgstrøm R, Salbu B, Gebremariam Z, Dadebo E (2014). Organochlorine pesticides and polychlorinated biphenyls in fish from Lake Awassa in the Ethiopian Rift Valley: human health risks. Bull Environ Contam Toxicol.

[75] Yohannes YB, Ikenaka Y, Nakayama SM, Saengtienchai A, Watanabe K, Ishizuka M (2013). Organochlorine pesticides and heavy metals in fish from Lake Awassa, Ethiopia: Insights from stable isotope analysis. Chemosphere.

[76] Yohannes YB, Ikenaka Y, Nakayama SM, Ishizuka M (2014). Organochlorine pesticides in bird species and their prey (fish) from the Ethiopian Rift Valley region, Ethiopia. Environ Pollut.

[77] Deribe E, Rosseland BO, Borgstrøm R, Salbu B, Gebremariam Z, Dadebo E (2011). Bioaccumulation of persistent organic pollutants (POPs) in fish species from Lake Koka, Ethiopia: The influence of lipid content and trophic position. Sci Total Environ.

[78] Melisie D, Damte T, Thakur AK (2016). Farmers' insecticide use practice and its effect on honeybees (Apis mellifera) foraging on onion flower in Adami Tullu district of Ethiopia. Glob J Pests Dis Crop Prot.

[79] Belie T Honeybee Production and Marketing Systems, Constraints and Opportunities in Burie District of Amhara Region, Ethiopia. https://hdl.handle.net/10568/721.

[80] Desalegn B (2014). Assessment of pesticides use and its economic impact on the apiculture subsector in selected districts of Amhara region, Ethiopia. J Environ Anal Toxicol.

[81] Mengistu ZM, Beyene JT (2014). Beekeeping in Ethiopia, a case of agrochemical uses in west Gojjam zone. Bee World.

[82] Mekonnen S, Gebremichael B, Gela A (2018). Assessment of Honeybee Colonies and production status linked with the application of Agrochemicals in Ejere District, West Shoa, Oromia, Ethiopia. J Vet Med Res.

[83] Workneh A (2011). Identification and documentation of indigenous knowledge of beekeeping practices in selected districts of Ethiopia. J Agri Exten Rur Develop.

[84] Hussen A, Westbom R, Megersa N, Björklund E (2007). Selective Pressurized Liquid Extraction for Multi-Residue Analysis of Organochlorine Pesticides in Soil. J Chromatogr A.

[85] Daba D, Hymete A, Bekhit AA, Mohamed AM, Bekhit Ael-D (2011). Multi residue analysis of pesticides in wheat and khat collected from different regions of Ethiopia. Bull Environ Contam Toxicol.

[86] Mekonen S, Ambelu A, Spanoghe P (2014). Pesticide residue evaluation in major staple food items of Ethiopia using the QuEChERS method: A case study from the Jimma Zone. Environ Toxicol Chem.

[87] Zelelew D, Gebrehiwot H, Bezuyehu D (2018). Multi residue analysis of pesticides in pre and postharvest wheat grains in Misha Woreda, Hadiya Zone, Ethiopia. Afric J Pure Appl Chem.

[88] Mekonen S, Lachat C, Ambelu A, Steurbaut W, Kolsteren P, Jacxsens L (2015). Risk of DDT residue in maize consumed by infants as complementary diet in southwest Ethiopia. Sci Total Environ.

[89] Mekonen S, Ambelu A, Negassa B, Spanoghe P (2017). Exposure to DDT and its metabolites from khat (Catha edulis) chewing: Consumers risk assessment from southwestern Ethiopia. Regul Toxicol Pharmacol.

[90] Fesseha H, Aliye S, Kifle T, Mathewos M (2020). Chemical and drug use in dairy farms of Hawassa Town, Southern Ethiopia. Public Health Open J.

[91] Deti H, Hymete A, Bekhit AA, Mohamed AM, Bekhit AE (2014). Persistent organochlorine pesticides residues in cow and goat milks collected from different regions of Ethiopia. Chemosphere.

[92] Gebremichael S, Birhanu T, Tessema DA (2016). Analysis of Organochlorine Pesticide Residues in human and cow's milk in the towns of Asendabo, Serbo and Jimma in South-Western Ethiopia. Chem Publ.

[93] Letta BD, Attah LE (2013). Residue levels of organochlorine pesticides in cattle meat and organs slaughtered in selected towns in West Shoa Zone, Ethiopia. J Environ Sci Health B.

[94] Mulugeta E, Addis E, Benti L, Taddese M (2017). Physicochemical Characterization and Pesticide Residue Analysis of Honey Produced in West Shewa Zone, Oromia Region, Ethiopia. Am J Appl Chem.

[95] Food and Agriculture Organization (FAO) FAOLEX Database. Pesticide registration and control proclamation Number 674/2010. http://www.fao.org/faolex/results/details/en/c/LEX-FAOC169467.

[96] United Nations Environment Programme (UNEP) Status of ratifications of the Stockholm Convention (2001) Accessed [2020 September 22]. http://chm.pops.int/Countries/StatusofRatifications/PartiesandSignatoires/tabid/4500/Default.aspx.

[97] Ministry of Agriculture (MOA) (2013). Export rejection notification summary report. Ministry of Agriculture Ethiopia;.

[98] Ministry of Agriculture (MOA) (2015). Export rejection notification summary report. Ministry of Agriculture Ethiopia.

